# Polymer Waste-Based Highly Efficient Maleated Interfacial Modifier in iPP/SCF Composites—Some Notes on the Role of Processing in Their Thermal and Dynamic Mechanical Properties

**DOI:** 10.3390/polym15061527

**Published:** 2023-03-20

**Authors:** Jesús-María García-Martínez, Emilia P. Collar

**Affiliations:** Polymer Engineering Group (GIP), Polymer Science and Technology Institute (ICTP), Spanish National Research Council (CSIC), C/Juan de la Cierva, 3, 28006 Madrid, Spain

**Keywords:** short carbon fiber, polymer waste-based interfacial agent, compatibilizer, aPP-SASA, polypropylene, iPP/SCF composites, TGA, DSC, DMA, SIRM, FESEM

## Abstract

This work has a two-fold objective. First, it attempts to present the excellent efficiency of a maleated interfacial agent (obtained by the authors by using atactic polypropylene industrial waste) when used as interfacial additive in polypropylene/short carbon fiber composites (iPP/SCF). Second, in this paper, we pay attention to the role played by processing in the final properties of the composite. This work has been performed by considering the emerging crystalline morphologies generated by the different shear forces that the molten material suffers depending on the molding method employed. The interfacial agent analyzed here consists of an atactic polypropylene containing succinic anhydride grafts obtained through a chemical modification process performed in solution. It incorporates different types of succinic grafts, such as succinic bridges between aPP chains and backbone and terminal grafts (aPP-SASA) in its structure, and contains 5.6% (5.6 × 10^−4^ g/mol) of grafted polar groups in total. The adhesion of the polyamide SCF sizing and the succinic units is followed by Field Emission Scanning Electronic Microscopy (FESEM) and Synchrotron Infrared Microscopy (SIRM). However, the main objective of this work is the study of the thermal and the dynamic mechanical behavior of the materials of a series of both compression- and injection-molded samples to ascertain the enhanced interfacial interactions in the material and further comparison between the results obtained by both processing operations. Therefore, we detect improvements of 200% in stiffness and 400% in the viscous response of the same SCF content composites caused by aPP-SASA, depending on the processing method used.

## 1. Introduction

It is well known that short carbon fiber (SCF) reinforced composites are broadly used for their low density, fatigue and corrosion resistance, low thermal expansion coefficient, high strength, and many other superb properties. It is for this reason that SCFs are widely used in aerospace, automotive, fuel cells, and other applications with high-performance requirements [1,2,3]. Additionally, the demand for carbon fiber reinforced composites has increased notably due to the need for new industrial applications demanding high strength-to-weight ratios, such as eco-friendly vehicles, robots, energy devices, and so on [1,2,3,4,5,6,7]. Together with the actual circular economy and sustainability scenario, the aforementioned aspects make polyolefins highly competitive in most flourishing markets requiring organic-based materials (nearly 200 MT per year) [8,9,10]. In fact, polypropylene is one of the most used polymer matrices for this composite type due to its properties that can be tuned by the effect of the filler and the interfacial agents [11,12,13,14].

Moreover, even in the case of using expensive virgin SCF, the superb properties obtained and the ease of recycling of both materials, polypropylene and SCF, make this an attractive route. Thus, even the recovered SFC from manufactured parts of waste disposal sources (aircraft, navigation, road, and railway transportation) offers a great opportunity of having cheaper SCF obtained from continuous carbon fiber through adequate procedures and with excellent properties for being used in thermoplastic-based composites [15,16,17,18,19,20,21,22,23]. However, the aforementioned aspects depend on good interaction between the fiber and the thermoplastic. One problem of SFC is its inertness, which, together with the nonpolar nature of iPP, makes the interface weak [1,2,3]. This disadvantage can be minimized by the use of sized SFC and interfacial agents promoting stronger interfacial interactions. Therefore, when the final objective is the improvement of the interfacial interactions between the components in heterogeneous polymer-based material, employing interfacial agents (or compatibilizers) has been demonstrated to be one of the most valuable strategies [11,12,13,14]. The primary condition that the so-called interfacial agent must mandatorily observe is its chemical resemblance with the polymer matrix, wherein the interfacial agent is allocated jointly to a high degree of chemical affinity with the reinforcement used [11,12,13,14,24,25]. Considering the latter, using non-conventional interfacial agents (such as those derived from polymer wastes) is a great way to better understand the composite system as a whole [24,25,26,27,28,29,30].

It is important to remark that a significant number (most) of works in the literature use commercial compatibilizers when studying iPP-based composites. However, this fact limits their forehead research to the relatively low number of options in the market. Indeed, the authors have evidenced this fact elsewhere [11,12,13,14,24,27,28,29,30]. Jointly, these works using commercial compatibilizers based on isotactic polypropylene do not pay any attention to the fact that the amorphous phase of the iPP matrix must allocate the interfacial agent [24,29,31]. Consequently, an amorphous-based interfacial modifier (aPP-SASA) would have more mobility in the surroundings of the amorphous phase hosting the reinforcement. 

In this way, concerning the interfacial modifier, this paper shows the effect of a non-conventional interfacial agent synthesized in our laboratories from polymer wastes [32,33]. The superb efficiency of the latter (aPP-SASA) has been proved in the iPP/PA6 system by DMA and other techniques [27,34,35,36,37] and also for the iPP/SCF system over-compressed samples [24]. Furthermore, this additive incorporates different types of succinic grafts, such as succinic bridges between aPP chains and backbone and terminal grafts (aPP-SASA) containing 5.6% (5.6 × 10^−4^ g/mol) of grafted polar groups in total [32,33].

Then, it is essential to mention that compression molding may be helpful when the purpose is to check the role of the interfacial agent (whether efficient or not) but not to maximize the properties of the system as a whole. Therefore, this is why this work undertakes the study of the iPP/SCF/aPP-SASA system by comparing the shaping method, and a spectacular improvement in the final property is reached. Furthermore, the processing methodology follows conditions of the emerging morphologies, so the ultimate properties of the material are a very well-known basic fact but very often dismissed in the literature [38,39]. 

Accordingly, this work concerns a comparative and exploratory study on the effect of a triple-type grafted succinic anhydride atactic polypropylene (aPP-SASA) on the thermal properties together with the elastic and viscous response of a series of iPP/SCF composites (where the SCF are polyamide sized commercial available) on the final material obtained by considering two different (mainly in terms of fluid dynamics and shear forces implied) shaping methods such as compression and injection molding. Therefore, for this study, we have employed dynamic mechanical analysis, one of the more powerful characterization techniques for semicrystalline polymer-based systems, to the high sensitivity to changes at an interfacial level [40,41,42,43]. Thus, this comparative study on the efficiency of aPP-SASA in iPP/SFC composites has been planned over two significant and extreme iPP/SFC ratios (85/15 and 60/40) by identifying the four relaxation zones of the iPP matrix [24,28,31]. Additionally, a thermal gravimetric analysis (TGA) and dynamic differential scanning calorimetry (DSC) have been performed to further study the thermal stability and evolution of the crystalline content to support the discussion on the DMA relaxation spectra.

## 2. Materials and Methods

### 2.1. Materials

**Figure 1:**
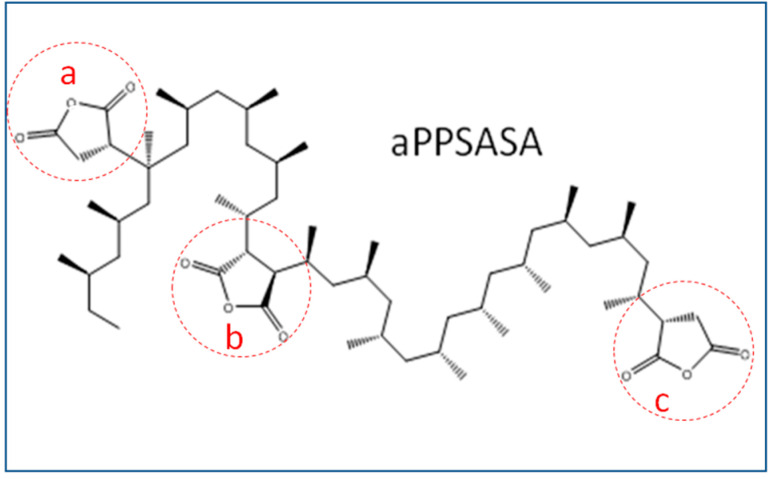
Waste origin interfacial agent containing side (a), bridge (b), and terminal grafts (c) onto atactic polypropylene chains.

As the polymer matrix, an isotactic polypropylene, Isplen 050 (ρ = 0.90 g/cm^3^; M_w_ = 334,400; M_n_ = 59,500; T_g_ = −13 °C), was supplied by Repsol. As the reinforcement, short carbon fibers (SCF) 4%, polyamide-sized, Toray 300 PA (ρ = 0.78 g/cm^3^; E = 231 GPa; σ = 3.8 GPa; Diameter = 7 µ) was supplied by Mitsui. The interfacial modifier used in this work was a succinic anhydride grafted atactic polypropylene grade (aPP-SASA) containing three different types of grafts and 5.6% of the total grafting degree obtained through a discontinuous process performed in solution by using an industrial by-product (atactic polypropylene) as the polymer reagent. The obtaining and characterization of aPP-SASA have been fully described elsewhere [32,33]. Figure 1 shows the chemical structure of the interfacial agent used. 

### 2.2. Sample Preparation

Considering this work is an exploratory and preliminary study, we have considered five different compounds. Therefore, the samples studied now obtained by two different routes (compression and injection molding) were a neat iPP (S0), two other composites with low SCF content (15%) in the absence (S1), and containing 1.5% of aPPSASA of the interfacial agent (S2), respectively, and other two with very high SCF content (40%) without (S3) and with 1.5 % of the interfacial agent (S4). Thus, it is well worth mentioning that this interfacial agent (aPP-SASA) was incorporated by replacing merely 1.5% of the iPP matrix in the composite. Table 1 compiles this information.

The compound was prepared as follows: iPP, SCF, and aPP-SASA were dry-blended. Afterward, this mixture was compounded into a counter-rotating twin-screw extruder Collin ZK-50 operating at 20 rpm and a temperature profile from 190 to 220 °C from the hopper to the die. Next, the pelletized compounds obtained were checked to ensure the reinforcement content in the master batch. After this process, the pellets were used alternatively to obtain both compressed plies and injected champions.

On the one hand, the compression-molded plies were obtained using a Dr. Collin press at 3.3 MPa, 180 °C, and then water cooled under pressure. On the other hand, conversely, we obtained injection-molded samples using a Margarit injection molding machine with a 210/200/200 °C temperature profile and a filling and compacting pressure of 850 and 600 kg/cm^2^, respectively. Thus, after a conditioning period of 48 h, at room temperature and 50% R. H., the compression plies and the injected samples were mechanized into prismatic specimens (20 × 5 × 2 mm) for the DMA analysis.

### 2.3. Characterization Procedures

**Figure 2:**
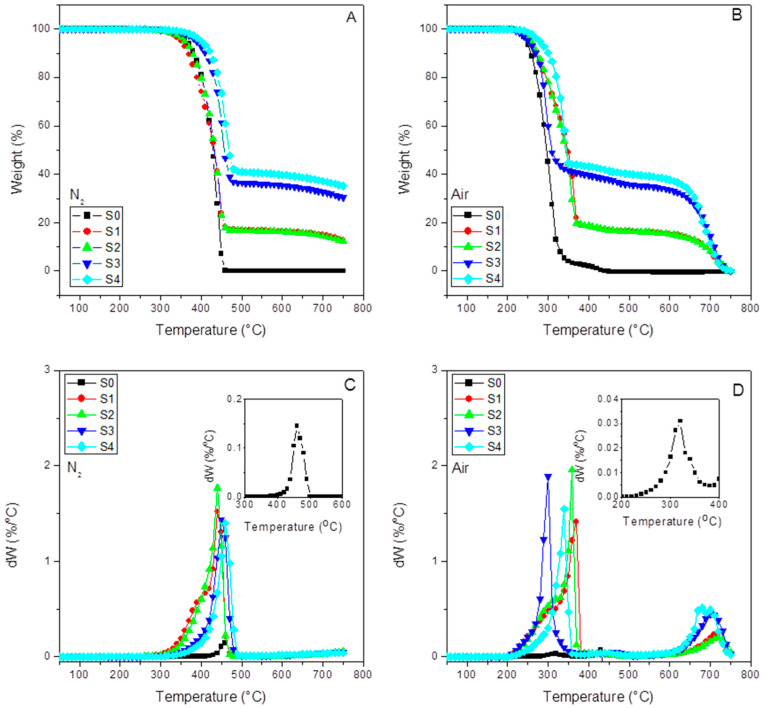
TGA and DTG curves for the indicated samples under inert (**A**,**C**) and oxidative (**B**,**D**) conditions. The DTG curves for S0 have been also included as inserts.

Thermogravimetry analysis (TGA) over the different granules obtained in the extrusion process was carried out in TAQ50 thermogravimetric analyzer. Experimental runs over samples of around 20 mg were undertaken by heating from 30 °C up to 750 °C at a heating rate of 10 °C/min under a nitrogen atmosphere (90 mL/min) to check the SCF dosing in the compound. In addition, the same experiment was conducted under an oxidizing atmosphere (air) to ascertain the thermal stability of the samples jointly with the fiber. During the experiments, DTG curves were also recorded. Figure 2 shows these thermograms of all the compounds under both atmospheres employed.

Scanning Electron Microscopy (SEM) micrographs were taken over the tensile fracture surface of previously DMA tested samples once gold coated by a sputter coater Emitech, K550x model. A Field Emission Electron Scanning Microscope (FESEM), Hitachy SU8000 at 1 kV, was used for the morphological observations.

A Nic-plan IR microscope coupled to a Magna System 560 Fourier transform infrared (FTIR) spectrometer (Thermo Nicolet, Madison, WI, USA) was used to observe and obtain FTIR spectra of over-compressed 10-micron thick samples. Images were collected at 32× magnification level. In all cases, the spectra were recorded at a spectroscopic resolution of 4 cm^−1^ and 32 scans, using synchrotron radiation rather than globar as an IR source (SuperACO, Lure, France). Therefore, it was possible to obtain spectra in 6 µm × 6 µm aperture frames, allowing us to visualize and map the carbonyl group assigned to amido group signals in the sample, including the surrounding of the matrix/fiber interphase. Data were recorded and analyzed using the Atlµs and Omnic E.S.P. software (Thermo Nicolet).

Differential Scanning Calorimetry (DSC) dynamically determined the thermal properties in a Perkin-Elmer DSC 7/Unix calorimeter. The calorimeter was calibrated with indium (T_m_ = 165.51 °C; ΔH_m_ = 28.45 J/g) and zinc (T_m_ = 419.51 °C; ΔH_m_ = 108.40 J/g) standards. Afterward, three aluminum crucibles per sample with around 15 mg each were tested under a unique dynamic melting run to maintain (non-erase) the previous thermal history of the samples for further comparison to DMA behavior. Therefore, a heating step from 50 °C to 200 °C at 10 °C/min, under a nitrogen atmosphere (30 mL/min), was run. Additionally, the reverse process was performed (cooling), and then a second heating scan was performed under the same conditions to observe the influence of the reinforcement and the interfacial agent once the thermal history of the sample was erased. The melting temperatures were obtained from the fusion peaks, and the crystalline content was determined from the fusion enthalpies by considering the real iPP content in the sample and the value of 209 J/g for a hypothetically fully crystalline polypropylene [44]. 

The dynamic mechanical analysis (DMA) was carried out under the tension mode by using a METTLER DMA861 by following ASTM 5026 standards requirements. They were machined into prismatic specimens (20 × 5 × 2 mm) from the compression-molded plies and the injection dogbone specimens and tested after a 48 h conditioning period at room temperature and 50% R. H. The DMA parameters, storage (E’), loss components (E’’) of the complex modulus (E*), and the damping or loss factor (tanδ = E’’/E’), were measured within the range of linear viscoelastic behavior of the material under a dynamic force of 12 N at 1 Hz frequency and 3 µm amplitude. The thermal scan was conducted from −30 °C to 140 °C at a 2 °C/min heating rate.

## 3. Results and Discussion

### 3.1. Thermogravimetric Analysis

Figure 2 shows the thermograms for each of the samples performed under inert (A) and oxidative atmosphere (B). At a glance, we can observe the correct dose of the SFC in the composites and the complete burning at 750 °C. Figure 2C,D (DTG curves) have also been included. 

Table 2 compiles the thermal parameters obtained from the TGA and DTG curves. The final residue results for all the samples, together with the temperatures at 0.5% (T_d0.5%_), 5% (T_d5%_), and 30% (T_d30%_) of weight loss, are compiled here. These parameters help estimate the thermal stability of the samples. In any case, we include the so-called heating resistance index (T_HRI_), a robust parameter to determine the thermal stability of a polymer-based material [45,46,47,48]. Therefore, under an inert atmosphere, we can observe that the presence of 15% SCF (S1) caused a decrease in T_HRI_ in respect to the pure iPP (S0), probably since the fibers at such amount enact a thermal degradation initiator effect. However, the presence of the interfacial agent in sample S2 increases the thermal stability till the values reach those close to that of S0. The latter suggests that the interaction between the amine groups of the SCF surface has reacted with the succinic grafts of the interfacial agent. We can observe a 13 °C increment in T_HRI_ and 19 °C for the one with aPP-SASA (S4) in respect to S0, and a 6 °C increment compared to the one without the interfacial agent (S3). These results can be explained by the fact that such extreme filler content (40%) highly affects the emerging morphology caused by a very constrained amorphous phase allocating the fibers (S3) and the interfacial agent in sample S4, together with the interaction between succinic groups and amine in SCF in the case of sample S4.

**Figure 3:**
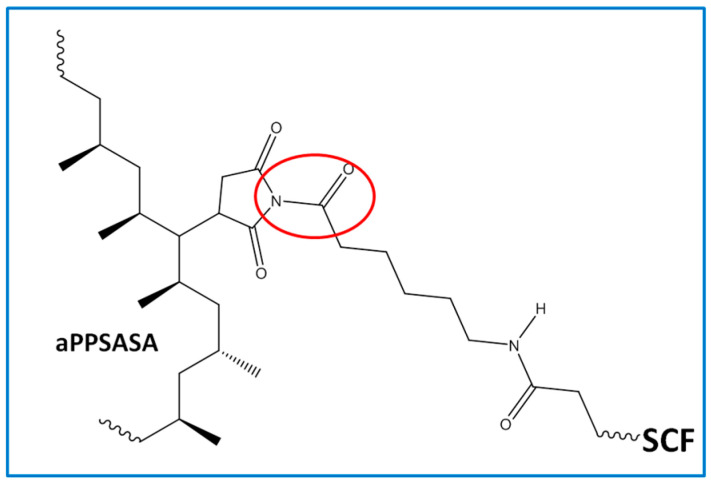
The chemical structure resulting from the reaction between NH_2_ groups of the SFC sizing and the succinic groups of the aPP-SASA.

Similarly, in the case of the oxidizing atmosphere (the actual scenario of a material under service), we can observe that the fiber increases the thermal stability of all the composites in respect to iPP. Furthermore, the presence of a-PP-SASA highly enhances this effect. At a glance, we see in Table 2 that 40% SCF (S3) increases T_HRI_ in respect to the neat iPP 5.3 °C and 21.1 °C if the interfacial agent is present (S4). Consequently, these data seem to confirm the noticeable effect of a mere 1.5% of aPP-SASA in the material’s thermal stability. All of the above-discussed is in full correspondence with the chemical reaction between the end NH_2_ groups attached to the SFC fibers and the succinic groups of the aPP-SASA, in agreement with previous findings by the authors of [24,27,39] (Figure 3). Notice that temperature values at 0.5, 5, and 30% of weight loss throw the same tendency as T_HRI_ under both atmospheres. It is also noteworthy to mention that under air, the values are not so different by the simple fact that because the polymer chain defects, double bonds, and so on, gain in importance as thermal degradation initiators.

Table 2 also includes the maxima temperatures (T_max_) from the DTG curves under inert and oxidative atmospheres together with a second temperature peak (T_SCF_) obtained under air ambient. The latter is assigned to the final SCF decomposition stage [49]. Additionally, under an inert atmosphere, we observe two shoulders in the S1 and S2 (compressed) samples, probably due to the polyamide decomposition [50] present in the SCF sizing. However, we do not see this effect in the injected champions (S3 and S4). In any case, this temperature is much higher than those used in the processing operations, so the sizing stays stable under these processing conditions.

As expected, the T_max_ shows a similar evolution as T_HRI_ (and the other reference T in Table 2), as discussed above. Therefore, no substantial different information about the thermal stability of the samples is obtained.

Curiously, the differences between the residue of the samples without and with aPP-SASA differ by approx. 4% (39.9% vs. 35.7% under nitrogen; and 38.9 vs. 34.9 under air for S4 vs. S3), entirely coincident with the SCF sizing content. One possible explanation is that the reaction between PA sizing and aPP-SASA produces a new molecule (Figure 3), suffering a charring process due to the succinic rings grafted to PA, similar to benzene bridges in other polymeric systems [50]. In any case, this effect observed for whatever the atmosphere used will be the subject of future studies.

### 3.2. FESEM Observations

**Figure 4:**
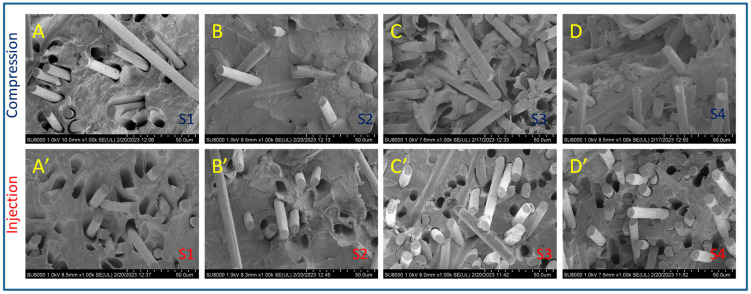
FESEM micrographs of tensile fractured surfaces of the iPP/SCF composites. Up: Compression-molded. Down: Injection-molded.

Figure 4 compiles de FESEM micrographs at 1000× magnification of all the composites studied in this work. These images’ primary purpose is merely to show the differences between the compressed and injected samples regarding fiber dispersion and orientation, together with the effect of aPP-SASA as an interfacial modifier. 

At a glance, we observe that the dispersion of the fiber is pretty good in all cases. However, the images of the compression-molded composites (up) show a less uniform filler orientation. The latter agrees with the flow dynamics imposed by the processing method used. Conversely, the corresponding injected specimens (down) offer a more oriented filler scenario by showing them almost perpendicular to the observation plane. 

Concerning the effect of the interfacial agent, we appreciate two different patterns depending on the processing method. In this regard, if we compare A (S1) and B(S2) samples, we see that S2 (with an interfacial agent) greatly diminishes the size of the gap around the fiber, suggesting a good interaction with the interfacial agent. We can conclude a similar result by observing C (S3) and D (S4) images but under a much more crowded scenario regarding fiber content. Note that the compressed samples present little pullout effect compared to the injected ones, confirming the transversal location of these in the specimen. It is remarkable that the print of a dragged fiber is fully transversal in the fracture surface (Figure 4B). Conversely, the injected samples (with fibers oriented parallel to the applied force to tensile break the sample, i.e., oriented along the injection flow lines) exhibited more holes than the applied forces due to the fiber pullout in all the cases since they are in the same direction. 

Observation of C’ and D’ curves allows us to conclude similar results. We maintain that including aPP-SASA (D’) drastically reduces the gap between the fiber and the polymer matrix compared with (C’), indicating a good interaction with the SCF. In any case, the effect of the interfacial agent is much more evident when the fiber content is low (A vs. B and, A’ vs. B’). It is important to remark here that S1, without aPP-SASA (A, A’), shows inverted conical holes that become cylindrical when the agent is present (B, B’). This effect may result from the DMA operation mode causing the fibers to cavitate if they are free enough, as is the case of a low-crowded composite. Conversely, the high fiber content in sample S3 does not permit this effect to be appreciated. In any case, the interfacial agent’s presence eliminates this effect for whatever fiber content we use.

**Figure 5:**
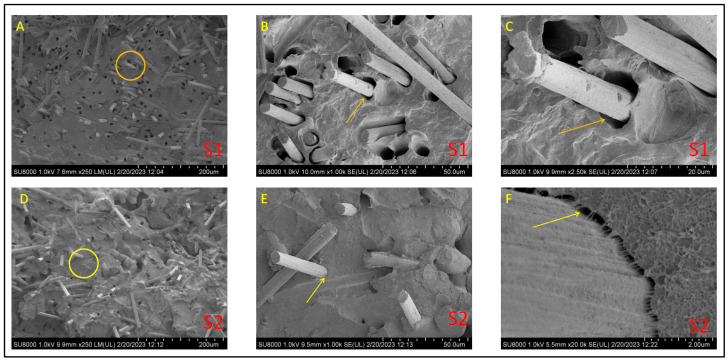
FESEM images taken from S1 (**A**–**C**) and S2 (**D**–**F**) compressed samples observed at different magnification levels.

Figure 5 shows images of compression-molded S1 (without aPP-SASA) and S2 (with aPP-SASA) composites observed at different magnification levels. We do that to visualize the interfacial agent’s effect more precisely. Therefore, a comparison of S1 and S2 at ×250 magnification levels (A and D) allows us to see (besides observing a good fiber dispersion) some differences in the image’s texture, the D image appearing smoother. 

Focusing on the yellow circle in Figure 5A, we appreciate a significant gap between the fiber and the iPP matrix without aPP-SASA. This fact is evident by observing the same zone (marked with a yellow arrow) at increasing magnification levels (1000× in Figure 5B and 2500× in Figure 5C), evidencing the absence of aPP-SASA in the system. The presence of the interfacial agent in images D, E, and F allows us to conclude the excellent efficiency of aPP-SASA. Moreover, by focusing on the yellow circle in image D (×250), we observe remarkable adhesion between the SCF and the iPP due to the action of aPP-SASA. Further, the observations at higher magnification levels (2500× in image E; 20,000× in image F) allow us to ascertain the fibrils linking the iPP matrix and the fiber, very probably due to the reaction between the fiber sizing and the interfacial agent giving rise to copolymers linking both phases. The image in Figure 5F agrees with the micromechanical model proposed elsewhere [35], already confirmed experimentally by the authors for the iPP/PA6/aPP-SASA system [27]. 

### 3.3. SIRM Observations 

It is well known that infrared (IR) spectroscopy is very sensitive to the heterogeneous nature of multiphase systems such as ours. The latter depends on the scale of the measurements and the size of the heterogeneous domains in the sample [51,52]. Therefore, when the subject is to identify reactions occurring at an interfacial level, it is important to detect that these interactions are happening at the interphase. For this reason, we have used SIRM to illustrate the evolution of the carbonyl absorbance of the amide groups (-CO-NH-) changing by the reaction with succinic units (Figure 3) in a composite containing our interfacial agent. 

**Figure 6:**
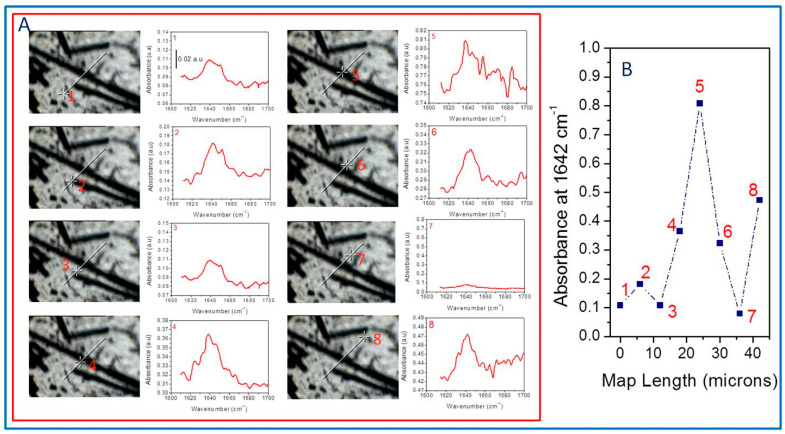
(**A**) SIRM images and spectra of the indicated samples obtained from S4 over-compressed specimens. (**B**) Evolution of the absorbance values for amido groups from SIRM image mapping.

Figure 6 compiles the FTIR spectra obtained every six microns along a representative line in the sample, corresponding to an over-compressed S4 specimen. In this way, we have eight different spectra with different absorbance intensities at 1642 cm^−1^ that allow us to follow the evolution of the signal across the sample. Figure 6A plots spectra identified with an image and a number for each point in the specimen wherein the spectra are collected. At a glance, we observe that the absorbance intensity depends on the place in the sample we obtain the spectra, which are very different when they are close to the fiber, suggesting the reaction between aPP-SASA and the –NH_2_ groups of the SCF sizing. 

Point 1 (far from the fiber but with some little fractured SCF in the surrounding) shows a lower signal than point 2 (obtained at the extreme of the thread, where the sizing is poor). Point 3, located slightly out of the fiber edge, throws values similar to point 1. Point 4, close to the matrix–fiber interface, significantly increases the signal. Consequently, the intensity sharply increases at point 5 (on the fiber surface plane), while point 6 provides an absorbance similar to that of point 6 (points 4 and 6 are almost equivalent in this map). Conversely, point 7 (far from the fiber influence and so with the absence of polyamide) abruptly decreases the intensity of the signal, while point 8 increases the absorbance intensity at 1642 cm^−1^ due to the combined effect of a new SCF and a fiber fraction. All the described aspects correspond with the polyamide sizing in the SCF and the reaction with the interfacial agent (aPP-SASA), similarly as in iPP/PA6 blends [27,36,50]. We have compiled all these data in Figure 6B.

### 3.4. Thermal Behavior and Crystalline Content

**Figure 7:**
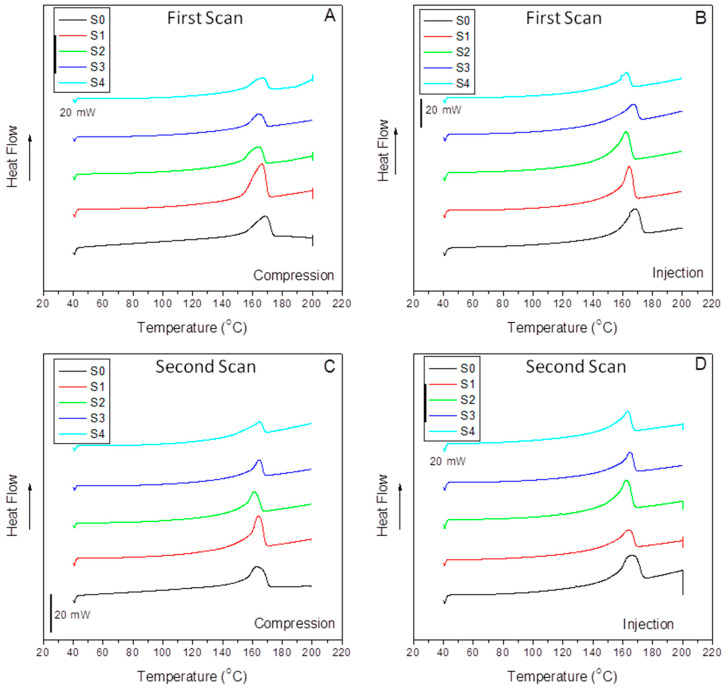
First and second DSC heating scans of the indicated samples under the compression-molded (**A**,**C**) and injection-molded (**B**,**D**) thermal histories.

Figure 7 compiles the DSC dynamic thermograms of all the compression-molded (A, C) and injection-molded (B, D) samples studied in this work without erasing the previous thermal history of the material (A, B) and after a second heating scan (C, D). The latter is performed to correlate to the DMA parameters, measured directly from the samples as molded. At a glance, we can observe a similar evolution concerning peak temperatures and differences in peak symmetry when comparing the compression to the injection origin samples. Table 3 lists the fusion temperatures, the enthalpies (considering the absolute amount of polymer in the composite), the crystalline contents, and the differences in crystalline content between compressed and injected samples. 

It is interesting to mention the similar values of the fusion peak for almost all the samples indicate that the crystalline cell has not suffered changes by the effect of the filler, the interfacial agent, or the molding method used, at least under our processing conditions. However, some differences of around 4 °C below are detected for the composites with 40% SCF under compression (S3) and injection (S4) samples, probably indicating the emergence of more imperfect crystals. At a glance, we observe that the crystalline content of the injected specimens is lower than that obtained in compression molding. This fact is related again to the elongation flow typical of injection molding that causes more constrained crystal/amorphous phase domains and lower crystalline entities, as evidenced by the broad left arm of the DSC curves in Figure 7B. 

In any case, the most exciting aspect is the vast differences between the crystal content of the S0 and S1 samples and S1 and S2, respectively, in the case of the compression-molded specimens and the S3 and S4 samples in the injection-molded ones. Here, we must pay attention to the effect of the interfacial agent and the impact of the processing method. The differences between the filled composites without and with the interfacial agent can be observed in Table 3. Thus, in the case of the compressed samples, a tremendous nucleating effect of the sample with 15% SCF composite (S1) evolving to a much lower crystalline content by the action of aPP-SASA (S2) is observed. This observation is strongly related to the role of the interfacial agent through the reaction of the amine groups and the succinic groups in a little constrained amorphous phase of the S1 sample due to the mild processing operation and the low SCF content.

Conversely, in the case of the much more constrained amorphous phase allocating the filler and the interfacial agent, the effect is just the contra ry. Thus, we observe a decrease in the crystalline content due to the aPP-SASA effective reaction with the fiber sizing. In the case of a more strenuous processing step (in terms of shear forces and elongational flow), as is injection molding, the shear forces imply the presence of smaller size crystalline domains with a highly constrained amorphous phase, as discussed in the previous section. The sharper (at the tip) fusion peaks in Figure 7B support this affirmation. As mentioned before, we can observe almost the exact value of 168.3 °C and 168.2 °C for the melting temperature of the neat compression-molded and injection-molded iPP (S0), obtained under homogeneous nucleation processes. However, in the case of the composites, with crystal growing under heterogeneous nucleation, we can observe a lower T_m_ caused by this effect for whatever the molding method used, except for the one with 15% SCFs when compression-molded. In any case, the differences in this parameter are high enough to evidence well-developed crystal morphology. Notably, these reordering processes across the iPP amorphous/crystal interface correlate well with the twin-screw extrusion mixing procedure used to minimize the fiber breakage because of the elongation flow component [24]. It is well-accepted that the elongation flow elements help the extended chain conformation of the polymer matrix during the processing steps, giving rise to a higher amount of tie molecules or interconnecting segments than when obtained under discontinuous mixing procedures where shear is the main flow component [24,53,54].

Further, this extended chain conformation even increases in the case of injection molding. Therefore, with the previous premise, it is clear that the tie molecules fraction results are a crucial factor in the material’s mechanical performance and is responsible for the loss of symmetry in the low-temperature arm of the melting peaks [47]. The sharper fusion peaks, compared to those of the compression-molded samples, evidenced the higher amount of tie molecules in the injection-molding samples (Figure 7). These DSC patterns and the T_m_ values (Table 3) are evidence of a well-developed α crystal morphology [24,53,55,56]. 

It is also well worth observing that the enthalpies (peak areas) offer quite similar values except for the compressed iPP/15%SCF (S1) and the injected iPP/40% SCF (S3) composites. The crystalline content of the compressed S1 appears 43.2% above that of the pristine compressed iPP, and 47.3% above that of the same 85/15 iPP/SCF/1.5% aPP-SASA composite compressed (S2). These results correspond with the growth of the transcrystalline region at the SCF surface. This effect can be explained by the low amount of fiber requiring a tiny PP amorphous phase to be imbibed and then the highest iPP supply to the transcrystalline regions growing around each thread [24,53,54,57,58,59]. Consequently, this effect plays a two-role role: First, the maximization of the total crystalline content of the iPP matrix, and second, the maximization of the non-free and highly constrained amorphous phase of polypropylene wherein the filler is allocated at the iPP/SCF interface [12,14,24].

Conversely, in the case of the compressed S3 sample (iPP/40%SCF), the fiber content is more than double, so the inter-distance between fibers is highly minimized, and the amorphous phase embeds any substance rather than increases the iPP. As a result, fewer PP chain segments are available to the crystalline and transcrystalline PP domains, and a lower crystalline content is detected compared to that of the compressed S1 sample (P.P./15%SCF).

It is clear that the crystalline content sharply decreases when the injected samples are compared to the compressed ones. In the case of a pristine iPP (S0) homogeneous crystallization regime [47,48,57,58,59], the decrease is close to 20%, in respect to the compressed sample. However, when the specimen is obtained under a heterogeneous crystallization regime [57,58], the samples with SFCs (S1 and S3), the decrease is 46% and 37%, respectively, caused by the combined effect of the flow dynamics and higher shear forces of the injection process. However, the compatibilizer (aPP-SASA) in samples S2 and S4 implies diminishing crystalline values close to the pristine iPP (S0). In any case, it is essential to remark that the presence of aPP-SASA causes the crystalline values to resemble notably that of the neat iPP (S0), and this effect is detected by whatever shaping method used in this work, evidencing the efficiency of the interfacial agent (aPP-SASA). 

Thus, the latter allows us to determine the interfacial role played by aPP-SASA able to liberate significant amounts of polypropylene chain segments that in the neat iPP/SCF composites (S1 and S3) are needed to imbibe the SCFs, and by reducing, on the one hand, the transcrystalline growth in the 85/15 iPP/SFCs composite, and on the other, by augmenting the overall crystalline content of the 60/40 iPP/SFCs system. That means the optimization of these composites by approaching each one to the crystal/amorphous ratio of the pristine iPP.

It is perhaps noteworthy to mention here that the relevant elongation flow component of the injection molding process implies a significant amount of the interconnecting segments that favor the extended chain conformations. The fraction of these interconnecting segments becomes a critical factor in responses dealing with the dynamic amorphous/crystal interface, considered to be the main one responsible of those reordering processes when the temperature approaches the melting temperature. The following section offers a discussion of the latter [57,58,59]. 

Table 4 compiles the values of the thermal parameters obtained for the second heating scan. At a glance, we observe a critical reduction in fusion temperature of neat iPP (for S0) of around 5 °C and low-content fiber composites (S1 and S2) between 2.8 and 5.7 °C for the injected and the compressed samples, respectively. However, the differences for the S3 and S4 show almost negligible changes in respect to the first scan, whatever the process followed.

It is important to remark that by focusing on compressed iPP (S0), we observe slightly crystalline content changes between the first and the second scan (42.6 v. 42.9). Conversely, the comparison between the scans for the injected sample (S0) allows us to observe that λ passed from 34.3% (first scan) to 40.7% (second scan), values similar to those obtained for the compressed samples. This does not occur for the other specimens, indicating that the only compound capable of erasing the previous thermal history is the neat iPP. Therefore, if we assume that a first heating scan erases the previous thermal history of the polymer phase, we must conclude that the crystalline content is not similar in both scans to the nucleating effect of the fiber. This phenomenon is modulated by aPP-SASA. This argument is supported by the fact that Δλ_m2_ exhibits much lower values than Δλ_m1_, indicating that the second DSC scan minimizes the differences between the samples obtained from such different processing techniques. Otherwise, it is important to remark on the significant decrease passing from nearly 60% in the first scan sample to close to 40% in the crystalline content of the compressed S1 sample after erasing the polymer thermal history. Since the compounds are hybrid materials (not only pure polymers), the effect of the processing technique is probably visible even in the second scan, and so the orientation of the fibers of the injection-molded samples might play a role. All the issues mentioned above deserve further investigation. 

### 3.5. Dynamic Mechanical Analysis

As mentioned above, it is a well-known basic concept in polymer science and technology that the processing operations abruptly affect the emerging morphologies and the distribution of the reinforcements (if any) in the obtained specimens. Unfortunately, however, this clear and intuitive concern is often ignored in many basic studies on polymer-based materials [38,39]. For this reason, the authors attempted to demonstrate, in an illustrative manner, the ways in which the final processing operations are the ones that decide whether a polymer-based material may be helpful or not. 

The study of polymer-based materials under cyclic mechanical and thermal conditions (DMA) permits the determination of both their elastic and viscous behavior separately. Therefore, we obtain a complex modulus, E* = E’ + iE”, with an in-phase response (Storage Modulus, E’) and one out-of-phase modulus (Loss Modulus, E”), and the damp factor (tanδ = E’’/E’). This technique provides a direct link between the chemical makeup of a material and its mechanical behavior, the difficulty centered in understanding the measured macroscopic property in terms of their microscopic origin [31,42,60].

We consider four different intervals in the dynamic mechanical spectra related to the relaxation and dissipation phenomena occurring when oscillating forces or deformations, as a function of temperature, are applied to whatever polypropylene-based material [24,31]. The first interval, located between −20 °C and −10 °C, is the zone where the polymer’s mechanical energy dissipation capabilities are lower due to being restricted to mere short segment motions. Therefore, variations of E’, E”, and tanδ with the temperature are insignificant. The second zone is between −10 °C and 40 °C, where the polymer’s glass (or β) transition occurs because of short-range but cooperative diffusion motions at the chain segment level associated with the amorphous fraction of the isotactic iPP of the polymer matrix in the composites. The third zone, between 40 °C and 80 °C, is characterized by the rubbery to elastic transition, typically exhibiting a plateau region in the E” curve for the neat iPP. In this gap, the rapid short-range diffusion motions strongly dependent on the molecular mass and the chain entanglement density occur. Here, it is usually detected the so-called α transition. Finally, in t fourth interval, 80 °C to 140 °C, the polymer chains may participate in dissipation modes linked to long-range configuration modes causing the flow once the softening state overpasses.

#### 3.5.1. Elastic Behavior: Storage Modulus Evolution

**Figure 8:**
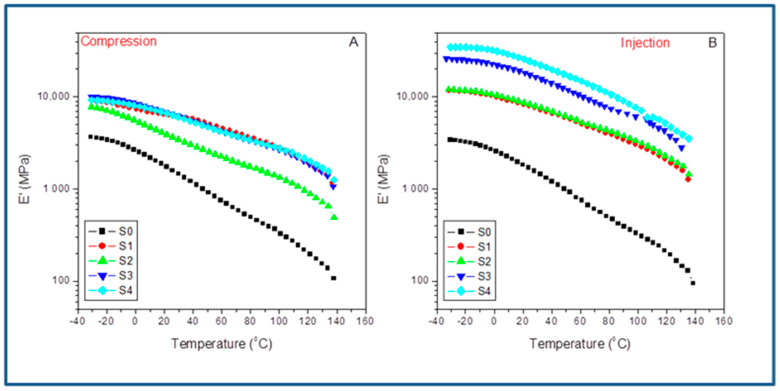
Evolution of the Storage Modulus with temperature for the indicated samples: (**A**) compression-molded; (**B**) injection-molded.

For a preliminary comparison of both molding methods, Figure 8 shows the evolution with the temperature of the storage modulus for the different compounds studied in the case of compression-molded samples (A) and injection set pieces (B). At a glance, we observe that the system’s stiffness is much higher in the case of the injection-molded filled composites than in the compression ones. Furthermore, all the curves are almost parallel but shifted upwards in the case of the injection samples. The latter can be explained as a consequence of the emerging morphologies and the preferential orientation of the SFC fillers all along the injection flow lines, which are not present in the compression-molded samples due to the radial molten flow; they may induce the random distribution of these fillers. This last aspect makes compression molding an option to merely evaluate the interfacial agent effect, if any, but no optimization of the ultimate performance of the material is possible [24]. This fact becomes a disadvantage when attempting to maximize the system’s overall behavior by the synergistic effect of both fiber orientation and the impact of the interfacial agent. Thus, although important, the changes observed for the neat iPP (S0) are very different by far from those regarded between the compressed and the injected composites. What is clear is that the crystal/amorphous reorganization possibilities change abruptly, depending on the molding technique used. Table 5 lists the storage modulus (E’) obtained from the indicated molded compounds obtained under compression (E’_C_) and injection conditions (E’_I_) for the indicated temperatures below and above the glass transition of the iPP matrix and the variations observed by comparing the two molding processes (ΔE’). It is worth noting that for the neat iPP (S0), the influence of the processing operations is low in all the temperature range transition (with a maximum of about 3% in ΔE’ at 25 °C). However, when the SCF is incorporated, the differences increase enormously depending on the molding method used. Behavior is much more different when comparing the composites with 15% of SCF without (S1) and with the aPP-SASA additive (S2). The processing strongly affects the final property, indicating that the injection molding always throws higher values (i.e., ΔE’ = 43.8% at 0 °C). When the interfacial agent is present (i.e., ΔE’ = 93.5% at 0 °C), it is located at even much higher values at different temperatures, significantly above the glass transition. More significant is the case of the composites with 40% of SCF content. The injected samples throw values (for instance) of ΔE’ = 169% in the case of the neat composite and ΔE’ = 281% when aPP-SASA is present, indicating the synergistic effect of both the fiber orientation and the interfacial agents in the stiffness of the system.

When comparing the mere effect of aPP-SASA within the same method, we can observe that this evolves conversely depending on the method used. The presence of aPP-SASA in the compressed specimen causes a decrease in the stiffness in all the relaxation zones, mainly in the case of the composites with 15% SCF (S1 and S2), butlower for the ones with 40% SFC (S3 and S4). Conversely, the action of aPP-SASA in the injected samples increases the storage modulus to a great extent for whatever temperature is considered (Table 4). The latter entirely agrees with the discussion in the previous section.

Consequently, the combined action of elongation flow and higher shear forces increases the system’s ultimate performance in terms of stiffness enormously. For instance, at −25 °C, the mere presence of 1.5% aPP-SASA increases E’ in the injected sample a 34.1%. Furthermore, we detected similar evolution in the other relaxation zones: 32.6% at 0 °C, 27.1% at room temperature, and 19.4% at 100 °C (even close to the softening state) (Table 4). Consequently, we can conclude that the aPP-SASA is extraordinarily efficient in iPP/SFC composites and that this efficiency is greatly maximized by the combined effect of the elongation flow and shear forces of the injection molding procedure, despite its lower crystalline content.

#### 3.5.2. Viscous Behavior: Loss Modulus

**Figure 9:**
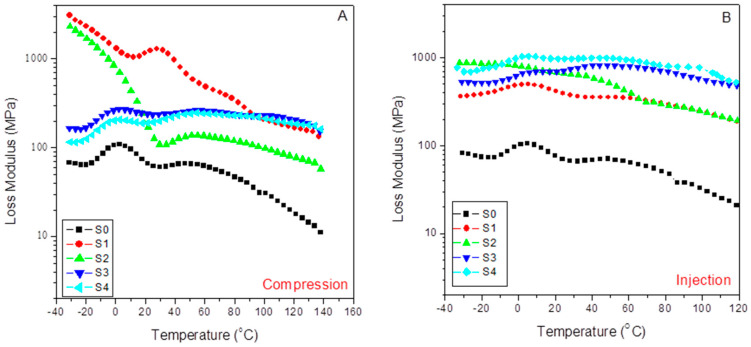
Evolution of the Loss Modulus with temperature for the indicated samples: (**A**) compression-molded; (**B**) injection-molded.

Figure 9 shows the evolution with the temperature of the loss modulus for the different compounds studied in the case of compression-molded samples (A) and injection-molded specimens (B). Thus, we observe a much higher viscous response in the case of the injection-molded composites than for compression ones, except for values lower than 60–70 °C in the case of the compressed composites with 15% of SFC (S1 and S2) with the independence of the content (or not) of aPP-SASA. The latter indicates that under these mild processing conditions, a tremendous nucleating effect is caused by the low amount of SFC (S1), and the stronger effect is caused by the presence of aPP-SASA (S2), as discussed previously. As mentioned before, this observation is strongly related to the role of the interfacial agent through the reaction of the amine groups and the succinic groups in a little constrained amorphous phase of the S1 sample due to the mild processing operation and the low SCF content. This type of behavior has already been reported elsewhere [24,28]. The latter can justify the emerging morphologies, and the preferential orientation of the SFC fillers all along the injection flow lines, which are not present in the compression-molded samples with a random distribution of these fillers.

Here, we observe that the changes for the neat iPP (S0) are comparable, namely the observed variations in respect to the injected samples that are lower than 20% and more typically lower than 10%. The latter indicates that the crystal/amorphous reorganization possibilities change less abruptly depending on the molding technique used if the material is just the neat iPP or incorporates SCF and aPP-SASA. What is clear is that all injection-molded composites throw higher values than the compression-molded composites, indicating that the capability of dissipation of energy is much more well balanced in the case of the injected composites than in the case of the molded ones, except in the above-indicated cases. The fact that changes in the glass transition temperature of the compounds can be clearly noticed may indicate that the combined effect of the filler, the agents, and the preferential orientation of injection molding modulated the values for this parameter. This aspect will be indicated when studying the evolution of the damp factor in the next section.

Table 6 lists the loss modulus (E’’) obtained from the indicated molded compounds obtained under Compression (E’’_C_) and Injection conditions (E’’_I_) for the indicated temperatures below and above the glass transition of the iPP matrix and the variations observed by comparing the two molding processes (ΔE’). In the pristine iPP (S0), we follow that the influence of the processing operations is relatively low compared with that observed for the filled samples, with a maximum of 19.9% in ΔE’’ at −25 °C. However, when the SCF is incorporated, the differences increase enormously depending on the molding method used. The sharp decrease in this parameter (−85.2%) observed in the viscous response for the S1 sample (15% SFC) diminished by the influence of aPP-SASA (S1), probably due to the reaction between the NH_2_ groups of the SFC sizing and the succinic groups that strongly alter the amorphous/crystal dynamic interface at such a low SFC content. This effect is also observed in the second relaxation zone, wherein the aPP-SASA can affect the short-range diffusion motion at a chain segment level. Thus, the processing strongly affects the final property. So, we conclude that the injection molding always modifies these values.. The processing methods’ action can augment the system’s viscous behavior till values well above 300% in almost all temperature ranges. In this way, 60/40 iPP/SFC with aPP-SA maintains a high value for stiffness in all cases (i.e., ΔE’’ = 498.4% at −25 °C; ΔE’’ = 363% at 0 °C; ΔE’’ = 405.0% at 25 °C; and ΔE’’ = 273.7% at 100 °C). The latter indicates the synergistic effect of both the fiber orientation and the interfacial agents in the viscous response of the system.

In addition, we can observe that the viscous content evolves differently by maintaining the molding method used but incorporating the interfacial agent. In the compressed specimens with 15% SCF (S1 and S2), the compatibilizer decreases the viscous response with temperature (−20.4% at −25°C, −37.9% at 0 °C, −89.9% at 25 °C, and −52.9% at 100 °C). Similarly, this is the evolved pattern for the S3 and S4 samples with 40% SFC (−28.5% at −25°C, −21.8% at 0 °C, −18.6% at 25 °C, and −11.8% at 100 °C). However, these samples exhibit a much lower decrease in the viscous response except at the lowest temperature. The latter entirely agrees with the constrained scenario for the amorphous phase caused by the higher filler.

Conversely, the action of aPP-SASA in the 15% SCF injected instances increase the loss modulus more than twofold, excepting this parameter at 100 °C, wherein the values are almost coincident (Table 6). In the same way, when comparing the 40% SCF samples with and without aPPSASA (S3 and S4), we observe lower decrements in the viscous response of the system despite their lower crystalline content than the compressed ones (33.1% at −25°C, 48.3% at 0 °C, 45.4% at 25 °C, and 35% at 100 °C), indicating the significant effect of the interfacial agent, especially at service temperature. This finding is consistent with the relaxation mechanism described earlier by considering the efficient impact of the interfacial agent by incorporating an additional amorphous phase to the very constrained scenario caused by the elongation and shear forces of the processing method. To summarize, we appreciate that the combined action of the elongation flow and higher shear forces increases the system’s ultimate performance in terms of energy dissipation in the case of the injected probes. Consequently, we can conclude that the aPP-SASA is extraordinarily efficient in iPP/SFC composites, and this efficiency is greatly maximized by the combined effect of the elongation flow and shear forces of the injection molding procedure due to well-balanced crystal/amorphous phase-generated interfaces.

#### 3.5.3. Damping Factor

**Figure 10:**
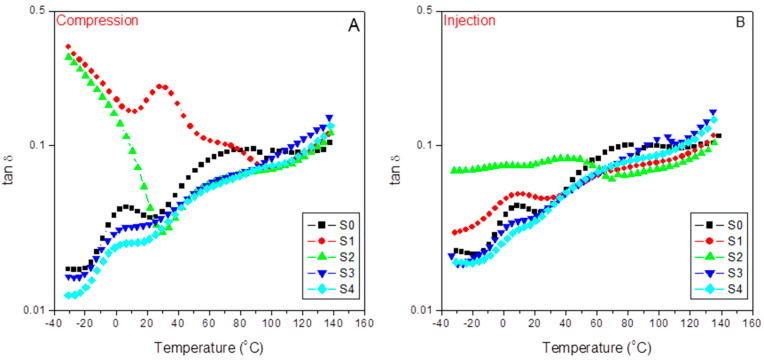
Evolution of the damp factor (tanδ) with temperature for the indicated samples: (**A**) compression-molded; (**B**) injection-molded.

Figure 10 shows the evolution of the loss or damping factor with temperature for the different samples in the case of compression-molded samples (A) and injection-molded samples (B). Since the loss factor is equal to the E”/E’, tanδ means standardizing the respective E” and E” variation rates for each material along the different relaxation zones. Therefore, increasing tanδ values means increasing variable rates on the viscous responses concerning those of the elastic ones and vice versa. The latter explains the neat iPP spectra (S0) appearing above that of the pristine and the modified 60/40 iPP/SCF composites (S1 and S2) independently of the molding processes used. This fact agrees with the expected decrease in the composite viscous character because of the presence of SCF, or, in other words, at the most significant packing density, as evidenced by almost overlapping between the two plots for S1 and S2 at the third temperature region assigned to the influence of the polypropylene amorphous/crystal interface domains of the material response. 

Moreover, it is important to note the significant increase in the pristine iPP (S0) response in this third region, where the α transition appears (76.6 °C and 78.2 °C, for the A and B plots) related to the metastable limit of this transition from the conformational disordered mesophases to the dominant polymorph. The latter means new dissipation routes available in the mechanical response of the PP matrix [59]. Additionally, all the plots tend to converge in the fourth relaxation zone due to dissipation flows across the iPP amorphous/crystal interface [24]. The filler, interfacial agents, and the different molding techniques also affect these transitions since they participate in emerging morphologies. In this sense, Table 7 compiles the values of these transitions as determined from the tanδ plots. 

At a glance, we observe an inverse evolution when comparing the injected and the compressed samples. Thus, for the 15% SCF tuned composite (S2), aPP-SASA liberates part of the iPP amorphous phase (imbibing the fiber) to begin this relaxation at a lower temperature and more in the case of the injected specimens. Nevertheless, on the other hand, the agent’s presence makes these diffusion motions more constrained due to the effective reaction between the amine groups of the fiber sizing and the succinic graft of the compatibilizer [24]. This fact is also evident in the case of the 40% SCF composites (S3 and S4) but less evident due to the previous more constrained scenario caused by the high filler content together with the much more strenuous processing conditions that injection molding means. A similar discussion applies in the case of the α transition, but relates now to the rapid short-range diffusion motions strongly dependent on the molecular mass and the chain entanglement density affected by both the presence of the interfacial agent and the emerging morphologies. 

Additionally, we ascertain (Table 8) that the loss factor response is higher in the case of the compression-molded filled composites than in the injected ones for values lower than those of the glass transition. The latter can justify the emerging morphologies and the preferential orientation of the SFC fillers all along the injection flow lines, which are not present in the compression-molded samples with the random distribution of these fillers.

Paying attention to the first and the second temperature relaxation regions is interesting. Here, the tanδ plots show that the viscous response dominates the dynamic mechanical behavior of the composites at the lowest packing density, which means the pristine and the modified composites are at a 85/15 iPP/SCF ratio (S1 and S2) that can be observed for both molding processes but is more noticeable in the case of the compressed one [24]. Both composites evidence maximized and seemingly continuous dissipation capabilities restricted to atomic and vibrational motions as they originate from the lowest temperature regions [59].

In any case, this effect disappears in the tuned composites (S2). It is remarkable that the E’’/E’ ratio stays almost constant (and much higher than all the other composites) in a wide temperature range (−30 °C to 50 °C), meaning that the presence of aPP-SASA stabilized the viscous/elastic ratio in these relaxation zones.

Alternatively, as tan δ is related to the impact performance of a material [60], the iPP/15%SCF/aPP-SASA system emerges as one attractive option to obtain materials with a stable impact performance in a wide temperature range coincident with the usual service conditions.

## 4. Conclusions

This preliminary work demonstrates the very efficient role of an industrial waste-based compatibilizer (aPP-SASA) in the final properties of iPP/SCF/aPP-SASA composites with very different and extreme fiber content. All the thermal techniques in this work detect this extraordinary effect of aPP-SASA by improving the thermal stability, modifying the amorphous/crystal interface balance, and consequently improving the system’s stiffness, viscous response, and varying the transition values of the polymer in the composites. In addition, FESEM and SIRM techniques support the existence of chemical bonds between the succinic units of the interfacial agent and the amide groups of the SCF sizing. Here, we preliminary compare two shaping procedures to ascertain not only the role of aPP-SASA in the system but the combined effect of this and the very different flow dynamics, shear forces, fiber orientation, etc., of the processing methods employed. The results suggest that the compression method is appropriate for testing the effect of the interfacial agents. However, when the objective is to maximize the final properties of the finished parts, a method implying a fiber orientation and higher shear forces shear offers much better properties. This fact suggests extending the study to broader compositional spectra using sDOE methodology. Once again, and to resume , we can undoubtedly establish that when we test a polymer-based material, we are jointly evaluating the material and the processing operations conducting the material to the solid state.

## Figures and Tables

**Table 1:** Identification of the samples studied in this work.

Sample	Composition
S0	Neat iPP
S1	iPP/15% SCF
S2	iPP/15% SCF/1.5% aPP-SASA
S3	iPP/40% SCF
S4	iPP/40% SCF/1.5% aPP-SASA

**Table 2:** Thermal parameters obtained from TGA analysis in Figure 2A,B.

		TGA						DTG	
Atmosphere	Sample	T_d0.5%_ (°C)	T_d5%_ (°C)	T_d30%_ (°C)	^1^ T_HRI_ (°C)	Residue (%)	T_max_ (°C)	T_shoulder_ (°C)	T_SCF_ (°C) (Air)
Inert (N_2_)	S0	302.2	362.4	413.8	192.7	0.1	458.6	----	----
	S1	280.3	348.5	407.6	188.1	16.5	442.9	391.3	----
	S2	290.5	356.5	415.2	191.9	16.1	441.4	391.2	----
	S3	310.5	386.3	442.3	205.7	35.7	452.3	----	----
	S4	318.3	399.2	454.1	211.7	39.9	460.2	----	----
Oxidizing (Air)	S0	218.0	245.0	283.0	131.2	0.0	315.0	----	----
	S1	222.3	253.2	314.1	142.0	15.0	372.5	----	717.2
	S2	223.7	255.4	315.2	142.4	15.3	361.4	----	718.8
	S3	219.8	256.6	293.4	136.5	34.9	297.1	----	704.7
	S4	237.5	278.4	332.4	152.3	38.9	341.1	----	695.8

^1^ T_HRI_ = 0.49[T_5%_ + 0.6 (T_30%_ − T_5%_)].

**Table 3:** Thermal parameters from DSC without erasing the previous thermal history fingerprint (first heating scan).

	Compression [24]			Injection			
Sample	T_m1_ (°C)	ΔH_m1_ (J/g)	λ_m1_ * (%)	T_m1_ (°C)	ΔH_m1_ (J/g)	λ_m1_ * (%)	Δ λ_m1_ (%)
S0	168.3	89.1	42.6	168.2	71.6	34.3	−19.5
S1	169.9	127.4	61.0	167.2	68.8	32.9	−46.1
S2	164.0	86.5	41.4	165.9	75.3	36.0	−13.0
S3	164.2	76.7	36.7	167.3	48.2	23.1	−37.1
S4	166.4	86.2	41.2	164.9	70.3	33.6	−18.4

* Crystalline content: λ_m1_ = (ΔH_m1_/209) × 100.

**Table 4:** Thermal parameters from DSC second heating scan.

	Compression [24]			Injection			
Sample	T_m2_ (°C)	ΔH_m2_ (J/g)	λ_m2_* (%)	T_m2_ (°C)	ΔH_m2_ (J/g)	λ_m2_ * (%)	Δ λ_m2_ (%)
S0	162.9	87.5	42.9	163.4	85.2	40.7	−5.1
S1	164.2	86.3	41.3	164.4	77.2	36.9	−12.8
S2	161.0	88.0	42.0	162.2	79.0	37.8	−10.0
S3	164.5	80.7	38.6	167.2	44.6	21.3	−44.8
S4	165.7	74.9	35.8	162.5	59.5	28.5	−20.4

* Crystalline content: λ_m2_ = (ΔH_m2_/209) × 100.

**Table 5:** Storage Modulus (E’) obtained from the indicated molded compounds obtained under Compression (E’_C_) and Injection conditions (E’_I_) for the indicated temperatures.

	T = −25 °C			T = 0 °C			T = 25 °C			T = 100 °C		
Sample	E’_C_ (MPa)	E’_I_ (MPa)	ΔE’ (%)	E’_C_ (MPa)	E’_I_ (MPa)	ΔE’ (%)	E’_C_ (MPa)	E’_I_ (MPa)	ΔE’ (%)	E’_C_ (MPa)	E’_I_ (MPa)	ΔE’ (%)
S0	3550	3560	0.3	2650	2600	−1.9	1600	1650	3.1	327	325	−0.6
S1	8850	11,500	29.9	7800	10,500	43.8	6200	7800	25.8	2800	2900	3.6
S2	7400	11,900	60.8	5400	10,450	93.5	3650	8400	130.1	1330	3300	148.1
S3	9990	25,800	158.2	8550	23,000	169.0	6650	18,100	172.2	2710	6450	138.0
S4	9000	34,600	284.4	8000	30,500	281.2	6400	23,000	159.4	2770	7700	178.0

**Table 6:** Loss Modulus (E”) obtained from the indicated molded compounds obtained under Compression (E’’_C_) and Injection conditions (E’’_I_) for the indicated temperatures.

	T = −25 °C			T = 0 °C			T = 25 °C			T = 100 °C		
Sample	E’’_C_ (MPa)	E’’_I_ (MPa)	ΔE’’ (%)	E’’_C_ (MPa)	E’’_I_ (MPa)	ΔE’’ (%)	E’’_C_ (MPa)	E’’_I_ (MPa)	ΔE’’ (%)	E’’_C_ (MPa)	E’’_I_ (MPa)	ΔE’’ (%)
S0	65.2	78.2	19.9	101.2	99.2	−1.47	62.0	68.8	11.0	30.7	31.1	1.3
S1	2500.0	370.0	−85.2	1305.0	485.8	−62.8	1250.3	337.8	−69.0	208.5	240.0	15.1
S2	1990.0	863.2	−56.6	750.3	810.2	8.0	126.4	668.3	428.7	98.2	242.3	146.7
S3	162.5	519.3	219.5	268.5	640.5	138.5	236.4	721.2	204.6	230.5	563.1	144.3
S4	116.2	695.4	498.4	205.2	950.2	363.0	192.5	971.9	405.0	203.4	760.2	273.7

**Table 7:** Transition temperatures for the indicated samples.

	Compression		Injection	
Sample	T_α_ (°C)	T_α_ (°C)	T_β_ (°C)	T_α_ (°C)
S0	6.3	76.6	7.3	78.2
S1	7.8	76.0	7.9	63.5
S2	2.1	63.9	1.6	69.8
S3	2.8	63.9	4.8	76.1
S4	1.3	63.5	4.3	68.7

**Table 8:** Damp Factor (tanδ) obtained from the indicated molded compounds obtained under Compression (^C^tanδ) and Injection conditions (^I^tanδ) for the indicated temperatures.

	T = −25 °C			T = 0 °C			T = 25 °C			T = 100 °C		
Code	^C^tanδ	^I^tanδ	Δtanδ (%)	^C^tanδ	^I^tanδ	Δtanδ (%)	^C^tanδ	^I^tanδ	Δtanδ (%)	^C^tanδ	^I^tanδ	Δtanδ (%)
S0	0.018	0.024	33.3	0.040	0.038	−5.0	0.037	0.042	15.5	0.085	0.097	14.1
S1	0.293	0.032	−89.1	0.179	0.049	−72.6	0.204	0.050	−75.0	0.075	0.081	8.0
S2	0.262	0.074	−71.7	0.148	0.077	−48.0	0.034	0.080	135.0	0.074	0.073	−1.3
S3	0.016	0.020	25.0	0.032	0.033	3.1	0.036	0.041	13.9	0.086	0.108	25.6
S4	0.013	0.020	53.8	0.025	0.028	12.0	0.030	0.038	26.7	0.079	0.089	12.6

## Data Availability

Not applicable.
